# Supplementary material for: Acceptance and compliance with micronutrient powder (MNP) among children aged 6–23 months in northern Nigeria
Source: PLOS Glob Public Health. 2022 Oct 17;2(10):e0000961. doi: 10.1371/journal.pgph.0000961 (PMC10022258; doi:10.1371/journal.pgph.0000961)
Supplement: S1 Dataset — (ZIP) [file pgph.0000961.s008.zip › S8_Dataset/In-depth Interviews.zip!/In-depth Interviews/080715_K_J_BZ_2_CG.pdf]

PHASE TWO (2) HOME-FEEDING TRIAL IN-DEPTH INTERVIEW WITH A MALE CAREGIVER AT HILINDE COMMUNITY, JEGA LOCAL GOVERNMENT AREA OF KEBBI STATE
ON 6th JULY, 2015

I: please I want you to describe people in your immediate family living in your house hold?

R: *To,* my immediate family in my house i feed them with food as you mate me in my farm, i am the one to take care of the whole and they are in good health conditions. We eat rice, *Tuwon dawa, tuwon shinkafa, Tuwon masara, Danwake,* etc.

I. Please could you tell me the gender of the baby that is taking NMP

R. Yes, the baby is female; her is Uwani, she is almost 19 month of age.

I: We have heard of food challenges for young children in this setting related to the seasons. What mechanism could be adopted in coping these food challenges?

R: If you look in to the current situation in our community here and our neighbouring communities we don't have enough or I can say availability of rice, *taliya*, vegetables, meat do to the lack of money

I: What mechanisms could be adopted in resist these challenges?

R: Mechanisms to cope the challenges is that we try as much as we could to stop selling our end of year produce and stored the produce it will go all year round.

I: I have heard of food challenges for young children in this setting related to the seasons. How could those challenges affect infant and young child feeding?

R: Once you don't have food available at you own reach, the children too will be affected but, their problems they may not understand the situation. Therefore you as a father you have work hard, run up and down to gate food for them to eat otherwise they will be crying for you throughout that night.

I. Uh

R. Yes, you have go outside to buy *masa, shikafa, shayi/tea, kosai, danwake,* sometimes have buy a*wara, shinkefa, kunu.*

I: One thing we heard in other interviews that caregivers often give their children 6-23 months is "tea". Could you please explain "tea" for me?

R: Tea we boiled water and eat it with *burodi*.

I. Uh

R. we boil water in the morning time, boiling of water, then letter we mixed with lipton and sugar we put milk and sometimes bounvita or milour.

I: Thank you for that explanation. Now I want us to talk about the MNP that you received. Could you tell me about your experience with the product?

R: My experience is that the MNP we gave to our baby we mixed it with *tuwo, kunu shinkafa,danwake, taliya.* The quantity of food we mix with MNP is the she can finish, we can't gave her the quantity that she cannot finish since we the capacity of food that she can consume because the person that bring the product to us that is Malam Ibrahim Jega he said we should mixed the product with the food that she can finished. It is good for every child to be given the MNP product because it is very good for their health, making the child very active, she is now stronger and healthier, she eat well, you will see her always fresh.

I: OK. I want you to talk more about the positive aspects of the experience with MNP.

R: So I told you that the MNP product to my child is good and I have seen a lot of improvement as well good changes in the healthier of my child, and she is now stronger and healthier and also good looking. If see her playing, running up and down you get to know that she is now stronger and healthier but before the child look inactive.

I: Please BABA, I want you to tell me a specific story relating to improvement in your child's health compared to before using the MNP.

R: We allot of experiences such as Polio, Tamowa has been treated and eradicated throughout our community. Before we seriously supper from polio and tamowa because we the parent hardly for us to accept that our children are suffering from tamowa. The only reason for not accepting tamowa was because is related to hungry issue and nobody accept that he cannot feed his family with enough food but, with the government interventions now on issue of tamowa and polio people our community really understood what they are stand for, and almost 80% to 90% of tamowa and polio is reduced.

I: Thanks you for your MNP story you gave to me. Could you talk about any general challenges you had during the MNP trial?

R: We work hard in explaining MNP value to our people but, they refused to understand, very few among others understand what we are saying about the MNP product. Honestly, up till now Uwani did not refuse the MNP product so don't have any challenges with the product, we are yet to notice any problems.

I: Tell me about any specific barriers that made it hard to use the MNP appropriately

R: Honestly, we don't have any problem concerning the use of MNP product because it is to use as we already have interest on the product, we thank Allah for your interventions.

I: Ok. Some caregivers mentioned that it was hard to store the MNP without it being taken by others or eaten by rodents. Could you please talk about your experience?

R: In my own case I don't have any problem with the product storage. If you want to see the product now, I will bring it now and it's well intact, in fact the product you gave to us is well protected.

I: Do you observed any side effect of the MNP on your child?

R: We did not encounter any problem with your MNP product.

I: Some mothers indicated that it might be hard to remember to use MNP. Did you have a similar experience?

R: No we don't have any problem like that because every day we gave one sachet to her, in fact we don't even jump a single day without giving her one sachet every day. I don't think we can forget to remember because we know it is very important for our child health.

I: Thanks for the information. What specific solutions could you suggest to address each of the barriers or challenges mentioned above?

R: Initially I told you that we did not encounter any problem with the MNP Product. But, may be the other people that received the MNP product from you in this community may

have something similar to that. We can go and ask, I all knew them very well even if you cannot ask, I can ask them on behalf of you, because I know they are in their farms or if you can wait for them to come back still there is no problems.

I: Thanks you, Let us now talk about the child's feeding and response to the MNP. Please talk about how you feed the MNP to the child usually.

R: Actually, we received your product and we are right now giving it to our child. We thank almighty Allah that gives us the opportunity to use the MNP product.

I: So what food do you use to mix with MNP?

R: We do mixed the MNP product with *fura, tuwo, shinkafa, and kunu*. Malam Ibrahim told us that we should use the food quantity that we know she can finished, even if she need more then letter that is after she finish taking the one mixed with MNP then you can add the food for her.

I: When do you usually give the MNP to the child?

R: As w are thought to gave her either in the morning and or in the evening time but, morning time is the best. That is why we are maintaining the morning time because whatever you do very early more especially feeding the better it is religiously and then even if I forget to remember to gave her in morning then we can gave her in the evening but, if we maintain evening some time we may forget to remember till tomorrow morning so we jump the whole day that means she miss one.

I: To what extent does the child likes or dislikes the taste of the MNP?

R: Uwani like the product very much because she never rejected the food mix with the MNP product that is my reason for saying she like it very much.

I: Ok. I want to know how, if at all, consumption of MNP impacted the child's diet.

R: Her food in takes have increased very high, compared with before she started consuming the MNP product, if buy *Bula* and slice it in a very small size mix it with groundnut oil you

will be very supersize the she will ate it. She ate everything you gave her, in fact she is now good in taking varieties of food, no matter what told you, you may not understand me until you so her eating by your selves.

I: What about her breast milk consumption?

R: She reduce suckling the breast milk of her mother, because she is takes varieties of food sometime if she go out with her elder brother for playing or to our niebourghs house she will stay long you will think we wean her but she is not weaned till she reached 21months of age. She is very active now and I so too many positive changes on her.

I: What about the varieties of foods consumed?

R: She ate everything you gave her, in fact she is now good in taking varieties of food, no matter what told you, you may not understand me until you so her eating by your selves. If you look at the child now you will see that she is becoming fresh, active, stronger and healthy but, before she is not like that.

I: In other communities, we noticed that sharing of the MNP occurred. Could you talk about any sharing of the MNP when you used it?

R: No we never give it to any one apart from Uwani that is who's MNP product is given for, not even her elder brother talk less of neiboughs and or friends in this communities. Malam Ibrahim told us clearly that the product is Main for Uwani alone, we should not share it with any one beside her, and as talking we really abide by what he said to us.

I: Please could you tell more on any sharing of the MNP due to food shortage during lean season?

R: Honestly, I have never heard any problem of food shortage in my house.

I: Please tell me whether the mother or father do ate out of the MNP as a snack

R: Not at all' she doesn't ate it. She gave it to the child alone as prescribed by you, one per day.

I: Tell me whether 60 sachets will be enough for 60 days.

R: Yes, 6o sachet is enough for 60 days. You know that whatever concerned you children related to health you must be serious about it so, we can't just play with the MNP product because we it is important to the body of our children. There once you have good storage of MNP so, you will not miss any one, 60 sachets is ok for me in 60 days.

I: Ok. Now that you are using the MNP, could you talk about how you perceive it?

R: My perception is that the MNP Powder is very easy to use. Just as the person who brought it explained, he said that it can be administered in the morning, afternoon and evening. But he recommended morning. So that, when one forgets to administer it in the morning, he/she can remember before the day runs out.

I: Ok. Do you see the MNP as food, medicine or something else?

R: Honestly I see it as medicine.

I: OK. Why?

R: Because the way that Malam Ibrahim introduce it to me , he said it is a medicine, prescribe it to me is almost the same way that the health personnel in our clinic use to prescribe the medicines for us when we are seek and because it helps in improving the health status of the children's.

I: If this product were cheaply on sale in your community, would you purchase it?

R: Yes, I can buy, because I know the product advantage, it is good for the children; it's making them stronger and healthier.

I: How much would you be willing to pay for a sachet?

R: Yes, I can buy it at the rate of 50 naira per sachet.

Because I sow the product positive effect on child meaning that I where we come from with baby and I know where we are right now so, the boy is doing the great. No matter how much

you fix on the product, to me is not costly because if you calculate the total amount of money I will spend when he is seek, it will higher than the amount of MNP product.

I: You are giving me great information. Now please tell me some ways that you believe would be effective for promoting MNP to caregivers in the community so that they would comply with using it appropriately

R: Honestly, when you want promote the MNP product is by telling people the advantage and disadvantages of MNP product, enlightenment campaigns, educating our people on how the MNP product should be given; how it should be use; its importance and positive and negative effect of the product on the children.

I: Are there other ways we can follow to ensure the caregivers use it appropriately?

R: You can only achieved appropriate use of MNP product by using the caregivers that have interest in using the product for their children and by the use house hold head of their respective house hold. These are people that can help the caregivers to make use of the product usage appropriately.

I: Thank you so much on that information. What people most influence your opinions and knowledge about feeding practices?

R: The people that influence my opinion on the feeding practice of the young children with MNP product was Malam Umaru, and Illo they were all their when Malam Ibrahim was explaining and introducing the MNP product to us in our community.

I: Ok. Who influenced your decisions regarding feeding your baby with the MNP?

R: You know I told you that the mother or the father are the one feed the children of under two years or the elder sisters or brothers too can feed the children. So my religion and the people around me are the one that influence opinion the feeding practice of the young children in my house.

I: Where would be good places to disseminate health information related to MNP?

R: The good place to disseminate health information related to MNP is in our village head palace. These well non place in which even a small boy of two years can show you the house of our village head and if he call on us we are all willing to go because he can't call us for nothing, ones he call us we know that is something good or something that is not good so that we can avoid it, still I said it we all respect that palace. There whatever good you want to disseminate or informed people of our community it has to be from there.

I: Thank you for that explanation. Who in your community do you trust the most for receiving messages about infant and young child feeding?

R: The person I can trust must in these community is our village head.

I: What forms of media do you prefer for receiving MNP messages? Brochure/Flyer/Posters/Radio/?

R: Yes, I know posters are very good information displayer for any king of information you want to send to us because majority of us cannot read or write but we all understand pictures, because is something that you can see and sometimes you can get detail information about the MNP, and radio.

I: What about radio?

R: Radio is the most important way of receiving and or send MNP message in our community. Radio is good everywhere in these community even if you don't have it someone close to you or in your house may have it, that why I said Radio is the best because we attached serious to it, once a serious message has been passed, we can take it as serious because allot of program are going own about farming other health related issues so we really appreciate radio very much.

I: What other ways?

R: The one I mention earlier is the only way and will be the best to receive or send MNP message in our community is using Radio.

I: Could you please tell me what ways would you prefer to receive education regarding IYCF and MNP?

R: I initially told you that it should be through the health personnel in this community and though our village head and our religious leader and other influential people in this community.

I: Thanks so much for that suggestions. Tell me about specific examples of other successful health education campaigns in this community.

R: Yes we have so many successful programs, some people in this community seem to be very serious on issues while they are not serious and some they are very serious. Therefore those that are very serious on some successful programs they are always the good achievers. For example on the onset of polio most of our people dislike the polio do our negative perceptions on polio that is to reduce the rate of birth in the north but, if you compare the birth rate now with 10 years back is on comparable. But *Alhamdullah* our people now understand the reason is to eradicate polio out of country and they have achieve that.

I: What are the best community channels to distribute the product?

R: The right channels to use in distributing the MNP is the village head and health personnel in our community, because the village head knows everybody in his community and we all know that he is honest and troth. So whatever you gave out to people, I assured you in as far as the village head concern the product will reach where you want the product to reach or intended people for the product.

I: Thanks sir. I want to hear some specific messages you think would effectively promote the product.

R: I have told you earlier that the village head with the help and town announcers, rigorous training, educating and enlightenment about the MNP product.

I: Please could I hear some specific message you think would effectively promote the MNP product?

R: The message i think you should be using the people with experiences, I those that use the MNP product they explain better than the people without having experiences.

I: Ok. What other specific messages could be used to ensure that it will be used appropriately?

R: Educating our people on how the MNP product should be given; how it should be use; its importance and positive effect on the children. If possible use our indigenous people to be explaining these product and use of female for their female counter part. If that can be achieved I assured you that the product will reach where you are not even expecting.

I: Thank you so much for that message. What specific messages could be used to promote its use but limit the sharing of it?

R: The message should be detail explanation on how it should be used and how important it is for the children. You should explain to caregivers that these is the target children for the MNP product and is main for them alone, so that you can limit MNP sharing among caregivers or friends.

I: Thank you so much. Finally, is there anything else you can share about your experience with this product, placement, price?

R: I am ok with the product; the price too is ok for me as i said earlier that 50 Naira per sachet.

I: What else would you recommend to develop an effective programme?

R: My recommendation is that the government should try as much as possible to provide enough of MNP product every one that need the product will gate it easily.

I: Thanks.
